# Unexpected Etiology of Intussusception in an Adolescent Patient

**DOI:** 10.1097/PG9.0000000000000342

**Published:** 2023-07-17

**Authors:** Yasir Alshareefy, Ali Alshareefy

**Affiliations:** * Trinity College Dublin, The University of Dublin, Dublin, Ireland; † MedCare Hospital, Dubai, United Arab Emirates

**Keywords:** intussusception, adolescent, clinical oncology, ileocecal valve, Burkitt lymphoma

## Abstract

Intussusception is a common cause of acute and subacute small bowel obstruction in children, young, and older patients; however, despite increasing awareness of the condition and the number of patients diagnosed with it across all ages, its clinical and diagnostic approach remains challenging. A 17-year-old girl attended our gastroenterology outpatient department complaining of a 6-month history of recurrent right iliac fossa pain associated with nausea and vomiting at times with no past medical history of note. Initial blood tests revealed a slightly raised CRP (9.1 mg/L) and a significantly elevated fecal calprotectin (>1000 µg/g). Computed axial tomography scan of the abdomen and pelvis revealed ileocecal intussusception with no evidence of small or large bowel obstruction. On subsequent colonoscopy a 5-cm mass protruding through the ileocecal valve was identified and multiple biopsies were taken for histological analysis, which confirmed a diagnosis of Burkitt’s lymphoma. The lesion was surgically resected and plans for adjuvant chemotherapy were discussed. The learning lessons to take from this case are to widen the list of differential diagnoses of unexplained recurrent abdominal pain to include intussusception and to actively rule it out with an appropriate diagnostic approach that addresses its potential malignant etiology across all ages.

## INTRODUCTION

Intussusception is a common cause of acute and subacute small bowel obstruction in children, young, and older patients attending emergency departments with obstructive symptoms like abdominal pain, nausea with or without vomiting. Despite increasing awareness of the condition and number of patients diagnosed with it across all ages, its clinical and diagnostic approach remains challenging. Intussusception is largely considered a condition of childhood with the highest incidence in the United Kingdom occurring in the fifth month of life (1). In children 90% of cases are idiopathic (2) and are easily recognizable due to the classical quartet of abdominal pain, bloody mucoid stools, abdominal mass, and palpable rectal mass in 70% and the classic triad of abdominal pain, bloody mucoid stools, and abdominal mass occurred in 32% (3). This differs in older populations where 90% of cases are secondary to a clear pathology such as carcinomas, polyps, and benign neoplasms, which often serve as lead point (2). Also, in contrast to childhood intussusception, older populations do not present with the classical symptoms and thus this poses a diagnostic dilemma with the most common symptoms being recurrent abdominal pain, vomiting, per-rectum bleeding (2). The main treatment options for childhood intussusception, upon diagnosis of this condition, is radiological enema techniques as a first line with studies reporting air enema to be more effective than liquid-based enema in the reduction of intussusception (4). The success of radiological reduction is dependent on a timely diagnosis and thus with delays in diagnosis there is an increased risk of requiring surgical intervention and bowel resection for management of childhood intussusception (5). The treatment options are more varied in regards to intussusception in older populations, as compared to children, due to the various underlying pathologies present in cases thus requiring varied management approaches. Our report describes the case of ileocecal intussusception, secondary to a Burkitt’s lymphoma of the ileocecal valve, in an adolescent patient. This report also describes a pragmatic clinical diagnostic algorithm on how to detect and confirm the diagnosis of ileocecal intussusception successfully.

## THE CASE

A 17-year-old female attended our gastroenterology outpatient clinic with a 6-month history of recurrent lower abdominal/right iliac fossa pain associated with nausea and vomiting at times. She was rushed to the local emergency departments on multiple occasions for assessment and analgesia. Medical assessments in visited emergency departments included physical examination, blood and urine testing, and abdominal imaging with plain radiograph and ultrasounds, which revealed no specific diagnosis. She was a healthy and active student with no personal or family history of note. Further enquiries about bowel habit, bleeding per rectum and weight loss were unremarkable. Initial tests including full blood count showed normal hemoglobin and white blood cells and a platelet count of 439 × 10^9^/L. C-reactive protein (CRP) showed only a mild elevation of 9.1 mg/L and there was a significantly raised fecal calprotectin level of >1000 µg/g.

**FIGURE 1.**
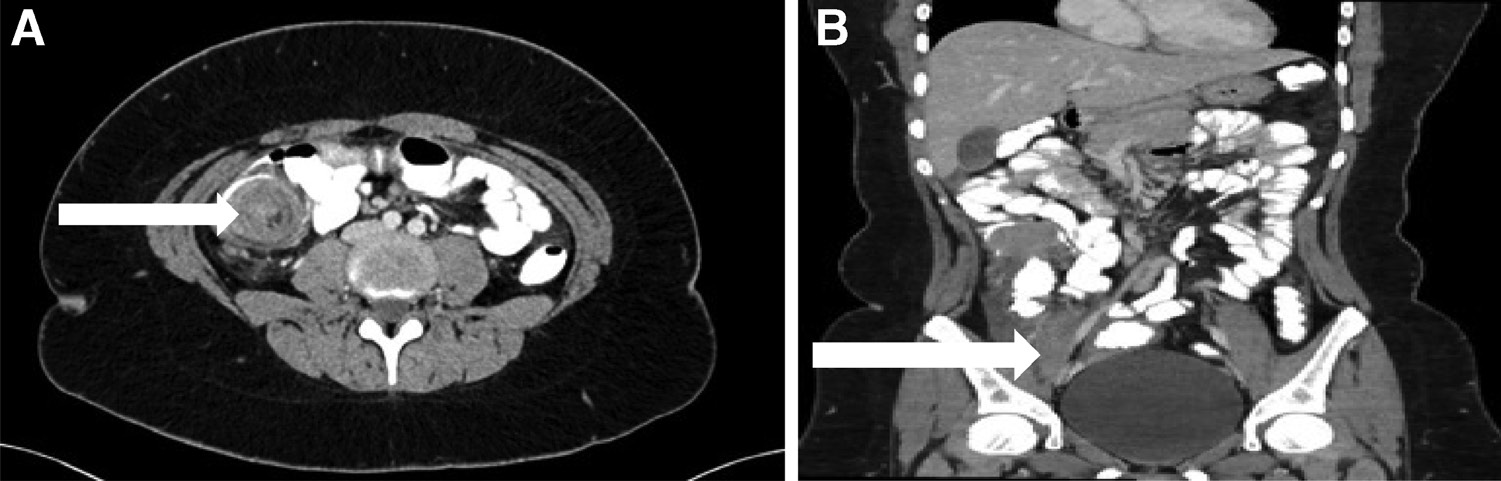
CAT scan abdomen and pelvis. A) Transverse section with arrow pointing to lead point of intussusception, (B) Longitudinal section with arrow pointing to ileocecal intussusception.

Given pain as her main presenting problem, she underwent a compound computed axial tomography (CAT) scan of the abdomen and pelvis with contrast which showed ileocecal intussusception with no radiological evidence of small or large bowel obstruction (Figure 1).

**FIGURE 2.**
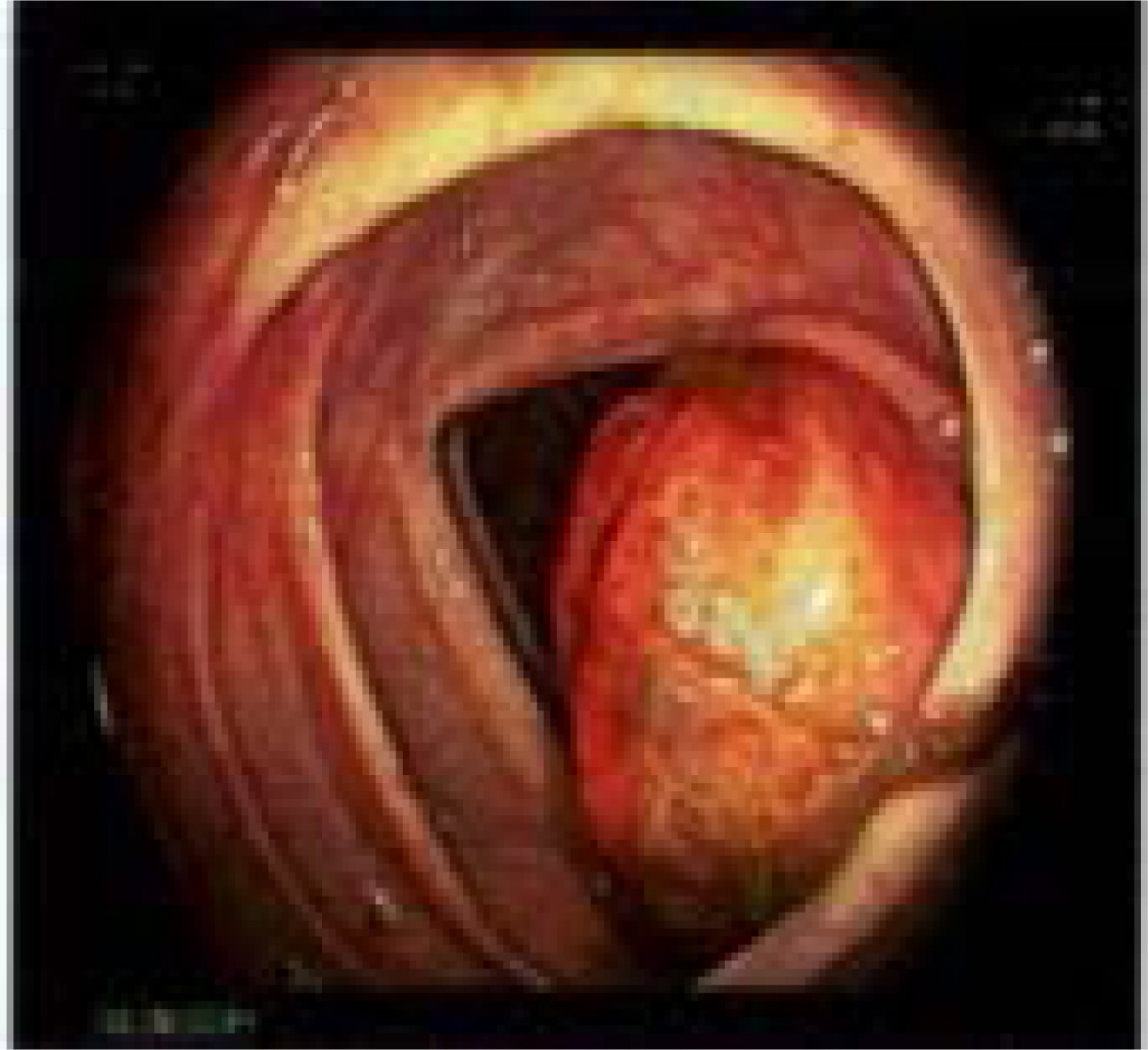
Endoscopic image revealing 5-cm mass in the ileocecal valve.

A colonoscopy (Figure 2) was then performed due to its potential for therapeutic intervention, diagnosis, and tissue sampling which revealed deformed cecal markings with a 5-cm mass protruding through the ileocecal valve, occupying all of the cecal pole, and causing severe local compression; however, intubation of the mass was achieved and lead to visualization of a normal but collapsed ileal lumen. No luminal lead-point-like mass or polyp could be seen. Multiple biopsies were taken, and a GI surgeon was called into the endoscopy room for direct inspection and discussion of a further plan of what appeared to be endoscopically an unrectifiable lesion.

The results of the endoscopic findings and options for further management were discussed with family and a plan of urgent surgical resection of the lesion, which should inform further plan of treatment according to histopathological examination of the surgical specimen was agreed upon. The lesion was surgically resected and sent for histopathological analysis.

**FIGURE 3.**
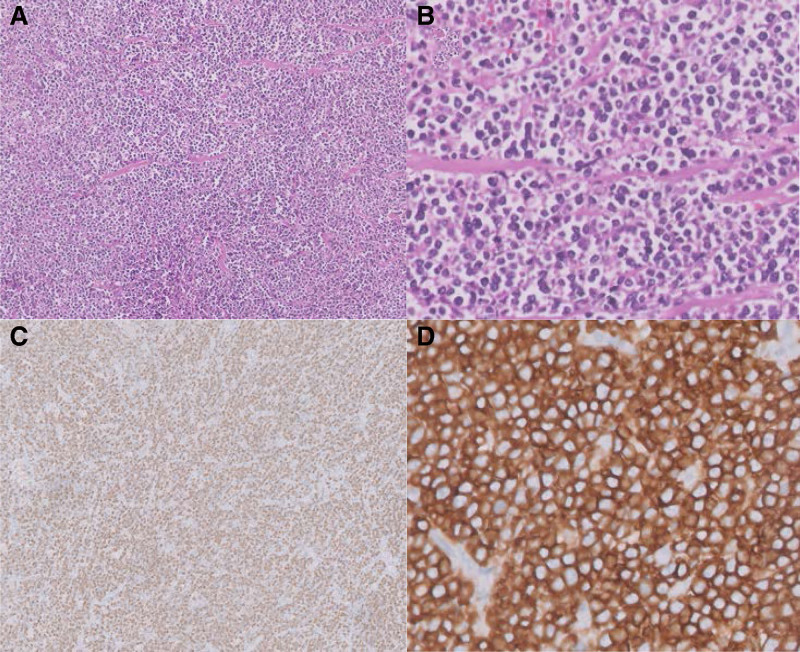
A) Atypical lymphoid cells at 20×, (B) Atypical lymphoid cells at 40×, (C) Immunohistochemistry studies showing strong CD20+ expression, and (D) Ki-67 index of 100%.

The histopathological analysis of the endoscopic biopsy and subsequent surgical specimen reported features of a lymphoproliferative state, suggesting a diagnosis of Burkitt’s lymphoma. Further immunohistochemistry testing demonstrated features of a B-cell immunophenotype, high-grade non-Hodgkin’s lymphoma favoring a diagnosis of Burkitt’s lymphoma (Figure 3). The patient was then referred to a hematologist for adjuvant chemotherapy which she has recently completed and has remained in good health.

## DISCUSSION

As we can see from Table 1, there is a distinct paucity in the literature regarding reports of intussusception secondary to Burkitt’s lymphoma in the adolescent population. Kulendran et al (6), report a similar case to that of ours, with a 15-year-old male presenting with ileocolic intussusception secondary to Burkitt’s lymphoma; however, there is a great difference in the timescale of symptoms with their patient presenting with a 3-week history of severe abdominal pain, vomiting, and diarrhea, compared with our case where symptoms of recurrent abdominal pain occurred over a period of 6 months. The majority of other cases published report similar diagnoses however in a much younger age cohort and thus our case provides a novel source of information on this topic in the adolescent age group. This report also describes a pragmatic clinical diagnostic algorithm on how to detect and confirm the diagnosis of ileocecal intussusception successfully; a history of recurrent abdominal pain ± nausea and vomiting in an adolescent should raise the suspicion of pediatric gastroenterologists for a possible diagnosis of intussusception. Ultrasound sonography and subsequent CAT scan of the abdomen and pelvis should be considered as primary imaging modalities for investigation. If ileocecal intussusception is detected on imaging, radiological reduction can be less successful in this clinical scenario and colonoscopy should be performed. While radiological reduction was traditionally used as the principal therapeutic option in children, however, by applying the same method in the adolescent age group can be less attractive from an efficacy point of view and may also be associated with a significant risk of missing an underlying malignant pathology. While colonoscopy has the potential for providing therapeutic relief via air reduction, it can also serve as an objective assessment tool for findings revealed in imaging studies, specifically CT scans. In addition, a colonoscopy may confirm the specific nature of the underlying pathology by sampling the lesions for histological analysis such as polyps or tumors. The learning lessons to be taken from this case are that the list of differential diagnoses of unexplained recurrent abdominal pain should be widened to include intussusception and to actively rule it out with an appropriate diagnostic approach that addresses its potential malignant etiology across all ages. We also hope this publication can raise clinical awareness for intussusception and its potential serious underlying pathologies in adolescents.

**TABLE 1.** Summary of Case Reports Describing Small Bowel Intussusception Secondary to Burkitt’s Lymphoma

Authors	Age	Gender	Type of Intussusception	Presentation	Management
Kulendran et al (6)	15 years	Male	Ileocolic Intussusception	3-week history of severe abdominal pain, vomiting, anorexia	Right hemicolectomy
Kennedy et al (7)	7 years	Male	Colocolonic Intussusception	2-week history of intermittent abdominal pain	Air contrast enema, left partial colectomy, adjuvant chemotherapy
Mehanna et al (8)	7 years	Male	Jejunojejunal intussusception	2-day history of abdominal pain and vomiting on background of intermittent colicky abdominal pain for 1 year	Laparotomy + end-to-end anastomosis of bowel + adjuvant chemotherapy
Angotti et al (9)	5 years	Female	Ileocecal-colic intussusception	1-week history of episodic spasmodic abdominal pain	Surgical resection + ileoileum anastomosis
Grajo (10)	7 years	Male	Ileocecal intussusception	1-week history of abdominal pain, 2-day history of constipation, 1-day history of nausea and vomiting	Laparotomy + ileocecectomy
Shah (11)	4 patients (median age 6.25 years)	2 male, 2 female	Ileocolic intussusception	3 patients: history of abdominal pain and umbilical lump for 1 week, 1 month, 2 months, respectively. 1 patient: recurrent intussusception with 2 previous admissions	3 patients: surgical resection + anastomosis + 2 cycles of adjuvant chemotherapy 1 patient: primary chemotherapy
Bieganska (12)	4 patients (4–14 years, average age 8 years)	4 males	Ileocecal intussusception in 3 patients Ileoileal intussusception in 1 patient	Symptoms: Right iliac fossa tenderness, nausea, vomiting Signs: bloody/tarry stool on examination	3 patients underwent laparotomy + resection + adjuvant chemotherapy 1 patient underwent laparoscopic surgery + resection + adjuvant chemotherapy In all 4 cases primary anastomosis was performed

## ACKNOWLEDGMENTS

The patient has given consent for the publication of this case report.

## References

[1] Samad L Cortina-Borja M Bashir HE. Intussusception incidence among infants in the UK and Republic of Ireland: a pre-rotavirus vaccine prospective surveillance study. Vaccine. 2013;31:4098–4102. 23871447 10.1016/j.vaccine.2013.06.084 PMC3988919

[2] Lianos G Xeropotamos N Bali C. Adult bowel intussusception: presentation, location, etiology, diagnosis and treatment. G Chir. 2013;34:280–283. 24629817 PMC3926485

[3] Ugwu BT Legbo JN Dakum NK. Childhood intussusception: a 9-year review. Ann Trop Paediatr. 2000;20:131–135. 10945064 10.1080/02724936.2000.11748122

[4] Applegate KE. Intussusception in children: evidence-based diagnosis and treatment. Pediatr Radiol. 2009;39:140–143. 10.1007/s00247-009-1178-9 19308373

[5] Kaiser AD Applegate KE Ladd AP. Current success in the treatment of intussusception in children. Surgery. 2007;142:469–75; discussion 475. 17950338 10.1016/j.surg.2007.07.015

[6] Kulendran K Choy KT Keogh C. An exceptional case of ileocolic intussusception secondary to Burkitt’s lymphoma: what variations are there in the presentation and management of those patients who approach adolescence?. Case Rep Surg. 2018;2018:1–5. 10.1155/2018/6251321 PMC6031204 30026997

[7] Kennedy K Rincon-Cruz L Weldon CB. Distal colo-colonic intussusception caused by Burkitt’s lymphoma. J Pediatr Surg Case Rep. 2022;86:102469.

[8] Hamad Mehanna K Ederaldo Queiroz Telles J Priscila Mauro D. Burkitt’s lymphoma presenting as jejunojejunal intussusception in a child: a case report. Int J Case Rep Imag. 2017;8:96.

[9] Angotti R Ferrara F Burgio A Garzi A Di Maggio G Meucci D. Intussusception in a child with sporadic burkitt lymphoma. JSAS. 2009;1.

[10] Grajo JR Kayton ML Steffensen TS. Presentation of ileal burkitt lymphoma in children. J Radiol Case Rep. 2012;6:27–38. 23365715 10.3941/jrcr.v6i8.1052 PMC3558269

[11] Shah H Tiwari C Khedkar K. Burkitt`s lymphoma presenting as intussusception in four children. Pediatric Oncall. 2016;13:71–73.

[12] Biegańska E Wolski M. Intussusception as a presentation of Burkitt’s lymphoma: a case series. . Med Sci Pulse. 2022;16:40–45.

